# Direct Cell–Cell Interactions in the Endometrium and in Endometrial Pathophysiology

**DOI:** 10.3390/ijms19082227

**Published:** 2018-07-30

**Authors:** Susanne Grund, Ruth Grümmer

**Affiliations:** Institute of Anatomy, University Hospital, University Duisburg-Essen, 45147 Essen, Germany; susanne.grund@uk-essen.de

**Keywords:** cell contacts, tight junction, adherens junction, gap junction, endometrium, implantation, decidualization, endometriosis, endometrial cancer

## Abstract

Cell contacts exhibit a considerable influence on tissue physiology and homeostasis by controlling paracellular and intercellular transport processes, as well as by affecting signaling pathways. Since they maintain cell polarity, they play an important role in cell plasticity. The knowledge about the junctional protein families and their interactions has increased considerably during recent years. In contrast to most other tissues, the endometrium undergoes extensive physiological changes and reveals an extraordinary plasticity due to its crucial role in the establishment and maintenance of pregnancy. These complex changes are accompanied by changes in direct cell–cell contacts to meet the various requirements in the respective developmental stage. Impairment of this sophisticated differentiation process may lead to failure of implantation and embryo development and may be involved in the pathogenesis of endometrial diseases. In this article, we focus on the knowledge about the distribution and regulation of the different junctional proteins in the endometrium during cycling and pregnancy, as well as in pathologic conditions such as endometriosis and cancer. Decoding these sophisticated interactions should improve our understanding of endometrial physiology as well as of the mechanisms involved in pathological conditions.

## 1. Introduction

Direct cell–cell contacts connect cells to each other to maintain cell polarity, stability and integrity. Moreover, they mediate selective paracellular as well as intercellular transport of molecules [1]. Due to these functions, they exhibit a considerable influence on tissue physiology, homeostasis, and tissue remodeling. In this regard, the endometrium is a special tissue, because in contrast to most other tissues, it undergoes considerable physiological changes and reveals an extraordinary plasticity due to its crucial role in the establishment and maintenance of pregnancy. Hormonally regulated cyclic changes in the tissue enable it to be transformed to a receptive state, which allows embryo implantation, attachment and invasion through the epithelium into the underlying stromal compartment [2,3]. During pregnancy, the endometrial stroma regulates trophoblast invasion and provides the blood supply for nutrition of the developing organism [4]. During these processes, the luminal endometrial epithelial cells undergo an epithelial-to-mesenchymal transition, whereas a mesenchymal-epithelial transition can be observed in the endometrial stromal cells [3,5,6]. These complex changes are accompanied by alterations in cell morphology, physiology and function concomitant with changes in direct cell–cell contacts to meet the various requirements in the respective developmental stage. Impairment of this sophisticated differentiation process may lead to failure of implantation and embryo development and may be involved in the pathogenesis of endometrial diseases.

In this review, we focus on the knowledge about the distribution of the different direct cell–cell contacts in the endometrium during cycling and pregnancy, as well as in pathologic conditions such as endometriosis and cancer. Results from human endometrium are correlated with findings from research with human endometrial cell lines and animal models.

## 2. Intercellular Junctions

**Figure 1:**
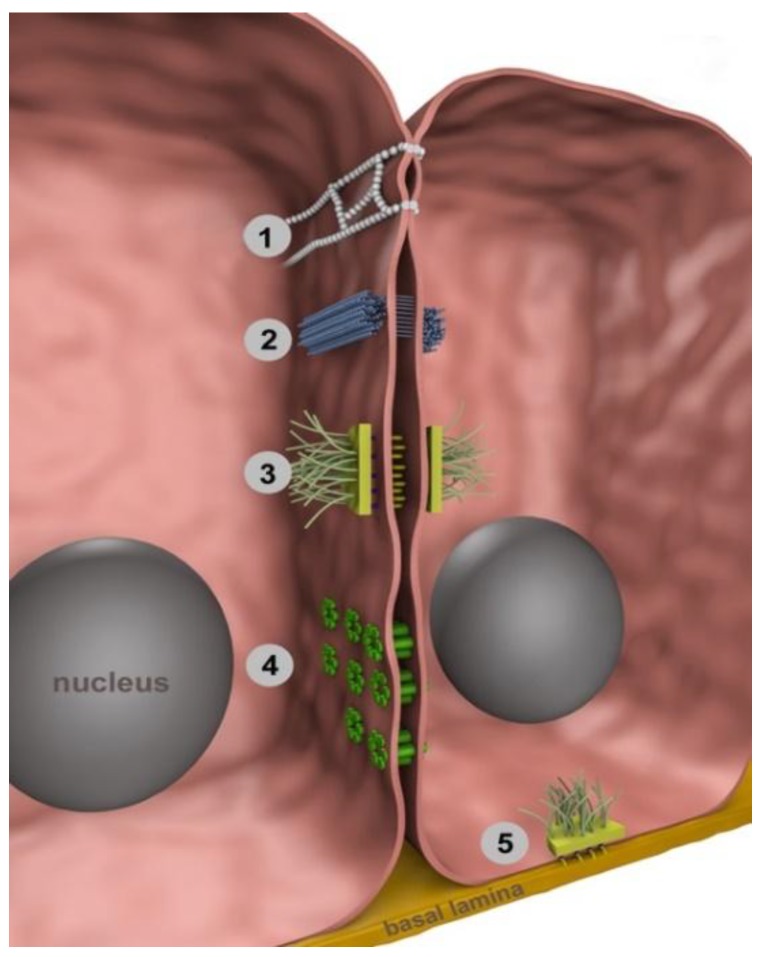
Localization of intercellular junctions. Tight junctions (**1**) are located at the uppermost part of the lateral cell membrane of two adjacent cells thus regulating paracellular transport between cells (gate function) and maintaining apicobasal polarity (fence function). Adherens junctions (**2**) and desmosomes (**3**) connect adjoining cells to each other. Meanwhile, adherens junctions are linked to intracellular actin bundles, desmosomal plaques are linked to intermediate filaments. Gap junctions (**4**) are intercellular membrane channels connecting directly the cytoplasm of adjacent cells, thus allowing the exchange of ions, second messengers and small metabolites. A gap junction channel is composed of two hemi-channels (connexons), each of which is composed of six protein subunits (connexins). Hemidesmosomes (**5**) connect intracellular filaments to the basal lamina.

Tight junctions, adherens junctions, desmosomes and gap junctions were originally identified by their morphological appearance in electron microscopy [7] and are localized mainly at the lateral membranes of polarized epithelial cells (Figure 1).

In recent years, there has been a considerable gain in knowledge about the molecules that contribute to the structure, function and regulation of those cell–cell junctions. Tight junctions are located at the uppermost part of the lateral cell membrane. They build up a selective barrier between the adjacent cells thus regulating and restricting paracellular transport between these cells (gate function). In addition, they maintain the strict organization of the plasma membrane of epithelial cells in an apical and a basolateral compartment (fence function) [8]. Meanwhile, a large number of proteins have been identified in this junctional complex. The key players in building up the barrier and fence functions are two types of transmembrane tetraspanins—claudins and MARVEL domain proteins like occludin—which form the core of the tight junction and are associated with cytoplasmic plaque proteins including ZO-1, -2, and -3 and MUPP1 linking tight junctions to the actin-cytoskeleton, as well as to the other members of the junctional complex. Of these, claudin proteins constitute the largest protein family, with 27 members identified in the mouse and 26 in humans [8,9]. In addition, claudins are able to recruit occludin to tight junctions [10]. Occludin is also a tetraspanin, acting as a paracellular barrier and maintaining cell polarity through interaction with ZO-1 [11] and ZO-2 [12]. The zona occludens proteins ZO-1, ZO-2, and ZO-3 can cross-link actin to the claudins and occludins, as well as to other cell–cell contacts such as adhesion junctions and gap junctions [13]. Moreover, they may recruit signaling components, thus playing a role in the regulation of gene expression [14]. In addition, the membrane associated junctional adhesion molecules (JAMs), which comprise three members, are discussed to be involved in junction assembly and paracellular barrier formation [8].

Adherens junctions and desmosomes are located just below tight junctions at the lateral plasma membrane. They keep neighboring cells together, thereby maintaining cell and tissue polarity. These adhesions are mediated by the transmembrane protein family of cadherins which include 20 classical members in the human [15], and which form homodimers in the intercellular space in a calcium-dependent manner [1]. The classical cadherins comprise E-cadherin, which is most abundant in the adherens junctions of epithelial cells, the neuronal N-cadherin, the vascular VE-cadherin, but also the desmosomal components desmogleins and desmocollins [16]. Besides their cell–cell connecting function, cadherins also bind directly and indirectly to many cytoplasmic proteins, particularly to members of the catenin family, like p120-catenin, beta-catenin, alpha-catenin, and to ZO-1, which in turn binds to actin filaments and microtubules [15], as well as to plakoglobin, plakophilins and desmoplakin in desmosomes [16]. ZO-1 also recruits ZO-2 into adherens junctions [12], thus establishing a connection to the tight junctional complex. In addition, it has been well documented that cell–cell adhesion is necessary for the formation of functional gap junctions [17], and that changes in the expression of adhesion proteins such as E-cadherin might contribute to an impaired localization of gap junctional proteins in tumor cells [18]. In epithelia, especially the association of E-cadherin and beta-catenin which may activate the Wnt-signaling pathway, seems to play a crucial role not only in cell adhesion, but also in a variety of other cellular processes and intracellular signaling pathways that control gene transcription [19,20].

In contrast to tight junctions and adherens junctions, gap junctions are intercellular membrane channels connecting directly the cytoplasm of adjacent cells. This intercellular communication allows the exchange of ions, second messengers and small metabolites, and thus influences cell differentiation and proliferation, as well as tissue development, homeostasis and morphogenesis [21]. A gap junction channel is composed of two hemi-channels (connexons), each of which is composed of six protein subunits named connexins (Cx) arranged around a pore. Up to now, 20 members of the connexin gene family have been identified in the human and 21 in the mouse genome. All connexins have four membrane-spanning domains forming the channel, two extracellular loops, a cytoplasmic loop and cytoplasmic N- and C-terminal tail segments which are involved in the regulation of channel function [22]. Gap junction channels comprising different connexins can exhibit different properties with regard to ionic conductance or intercellular metabolic coupling [21,23]. In addition, connexins may interact with cytoskeletal components thus regulating signal transduction processes [24,25]. For example, it has been shown that the cytoplasmic tail of Cx43 binds to ZO-1, ZO-2 and ZO-3, components of the membrane-cytoskeletal complex also associated with adherens and tight junctions [13,26]. Moreover, it is known that connexins may be located in the plasma membrane as undocked connexons allowing an exchange of molecules between the cytoplasma and the environment [27].

Summarizing these aspects, although the different cell–cell junctions comprise different proteins, there are similarities in the roles of specialized transmembrane proteins in forming extracellular adhesive contacts between cells, and intracellular links to the cytoskeleton and signaling pathways which may regulate gene transcription. Thus, all junctions, in addition to their classical function, may also be involved in processes such as proliferation and cell differentiation. Moreover, knowledge about the interaction between the different cell–cell contacts is continuously expanding and may have an impact also on endometrial physiology.

## 3. Cell–Cell Junctions in the Cyclic Human Endometrium

In the course of the menstrual cycle, both compartments of the endometrium, the epithelial and the stromal cells, undergo considerable hormonally regulated changes in preparation for embryo implantation. The luminal epithelium is transformed from a non-receptive to a receptive state, which allows adhesion and invasion of the trophoblast [3,28], while the glandular epithelium produces the components of the uterine fluid indispensable for the survival of the early embryo [29]. In parallel, the stromal cells differentiate to pre-decidual cells in preparation for trophoblast invasion [4]. Cell–cell contacts mainly have been described in the endometrial epithelial cells, providing and maintaining the polarity of the cells. In addition, they regulate the permeability of the epithelial cells controlling the exchange between the uterine lumen and the endometrium to create an environment which is appropriate for embryo implantation, and they are involved in the timely regulation to transform the luminal epithelium into a receptive state in which this physical barrier can be overcome by the blastocyst.

Already in 1982, freeze-fracture electron microscopy had revealed tight junctions in the luminal epithelial cells of the human uterus showing a decrease in junctional complexity from the late proliferative/early luteal phase to the late luteal phase of the menstrual cycle [30,31]. In parallel, the amount of desmosomes is reduced during this phase [32]. Using electron microscopy, Pan and co-workers also described tight junctions in the glandular epithelium of the secretory phase endometrium located apically at the basolateral membrane [33]. Moreover, the density of tight junctions was also shown to be reduced in the microvascular endothelium of the secretory phase endometrium compared to the proliferative phase [34].

With increasing knowledge about the composition of cell-to-cell junctions and the discovery of the high variety of proteins involved in these complex structures, knowledge about the changes in cell junction composition became more differentiated and complex in recent years. As members of the tight junction complex, up to now, claudin-1, -2, -3, -4, -5 and -7 have been described in human endometrial epithelial cells. Immunohistochemical staining revealed a localization of the claudins apically in the glandular epithelial cells while weak or no staining has been described in luminal epithelial cells [33,35,36,37]. In contrast, no staining for claudins was found in the endometrial stromal cells. These observations have also been described in vitro, since claudin-1, -3, -4 and -7 were detected in primary culture of isolated human endometrial epithelial but not in stromal cells [38]. Different results have been presented regarding the regulation of claudin expression throughout the phases of the menstrual cycle. While Gaetje and co-workers found no cyclic regulation of claudin-1 and -5 on transcript level by microarray analysis, the expression of claudin-3, -4 and -7 increased in the mid secretory phase compared to the proliferative/early secretory phase. However, this regulation could not be verified by immunohistochemical staining [36]. In contrast, the group of Sobel described a significant upregulation only of claudin-4 transcripts and an increase in claudin-1 and -5 protein in the secretory phase [35]. In contrast to that, other groups did not observe any regulation of claudin-3 and -4 throughout the menstrual cycle [33,37,39]. Interestingly, in women undergoing IVF treatment, the absence of claudin-4 in the presence of leukemia inhibitory factor (LIF) in the endometrium could be correlated with a 6-fold higher probability of successful establishment of pregnancy compared to samples that exhibited a strong claudin-4 and a weak LIF expression [40].

In addition to claudins, the tight junctional transmembrane protein JAM-1 was found at the basolateral membranes of the luminal and glandular epithelium [41] and also in a cytoplasmatic location in the glandular epithelium in both proliferative and secretory phase human endometrium [42]. As part of the cytoplasmatic component of the tight junctions, ZO-1 showed a distinct staining at the most apical part of the basolateral cell membrane of the endometrial epithelial as well as in endothelial cells [37]. For both proteins, JAM-1 as well as ZO-1, no change in the location or expression level could be observed throughout the different phases of the menstrual cycle [37,41,42].

Analyzing adherens junctions and desmosomes, a change in the localization of the cytoplasmatic plaque proteins desmoplakin 1 and 2, and the transmembrane cadherin desmoglein 2 from the apical pole of the lateral cell membrane in the proliferative phase to an evenly distributed pattern along the lateral cell membranes in the mid- to late luteal phase of the menstrual cycle has been described in the glandular epithelium of human endometrial tissue, while mRNA levels stayed constant. This redistribution was limited to the functionalis layer of the endometrium [37].

Immunostaining for E-cadherin and beta-catenin revealed a subapical localization at the lateral membrane of the glandular epithelial cells during the late proliferative and early luteal phase which disappeared during the mid-to late-luteal phase [37]. Also here, no significant changes in mRNA levels in could be detected throughout the cycle [37,43].

Gap junctions in the human endometrial epithelial cells were shown to consist of Cx26 and Cx32 [44,45,46]. An increasing intensity of Cx26 staining was observed in the uterine epithelial cells during the course of the proliferative phase, but it could hardly be detected in the secretory phase, including the receptive window. For Cx32, a weak expression could be observed at the basal portion of the epithelial cells which decreased during the receptive phase [44], whereas other studies demonstrated an increase of Cx32 in the early secretory and a decrease in the late secretory phase [46]. In contrast to tight and adhesion junctions, gap junctions are also present in the endometrial stromal cells. These channels are composed of Cx43. Like the endometrial connexins, the level of the Cx43 protein in the endometrial stromal cells has also been shown to decrease during the secretory phase [44]. However, while these publications point to a strong reduction of stromal intercellular communication during the receptive phase, others described an upregulation of Cx43 protein in the secretory phase [47].

Summarizing these findings, the amount and localization of the different junctional proteins change throughout the cycle, especially in the epithelial compartment, due to the hormonally regulated transformation of endometrial function. These changes are summarized in Table 1. Controlling the permeability of the uterine epithelium to establish an optimal milieu for the developing embryo and regulating endometrial receptivity to allow or prevent embryo implantation might be crucial functions of junctional components in the uterus. Though the morphology of the tight junction network gives an indication of their function in the epithelium, the claudin content is the parameter which ultimately determines the permeability characteristics [35,48]. Thus, the combination and ratio of the different claudins may be a key factor controlling embryo implantation. In contrast, up to now, only few junctional proteins have been described in the endometrial stromal cells. It is known that these cells express Cx43, but the findings about the cyclic changes are contradictory. However, the stromal cells undergo complete decidualization only during pregnancy. Here, the decidua plays an important role in embryo implantation and development. Thus, the amount and distribution of junctional proteins may considerably change during the implantation process.

## 4. Hormonal Regulation of Endometrial Junctional Proteins

The findings described above indicate that some of the junctional proteins are regulated throughout the menstrual cycle. To gain more detailed insight into this hormonal regulation, experiments in animals as well as in vitro have been performed.

A direct influence of hormonal stimulation on the structure of tight junctions has been described in a freeze fraction study in ovariectomized rats. Application of estrogen resulted in an apical shift of these junctions at the lateral epithelial cell membrane, whereas additional application of progesterone led to their extension down the lateral membrane [49]. Looking at the junctional components, ZO-1 was present nearly throughout the course of the cycle in the rat uterine epithelial cells, but was co-localized with occludin only during the estrogen-dominated proestrous phase, while occludin was absent in tight junction structures during the other phases of the estrous cycle. In parallel, a change in localization of claudin-1, -3, -5 and -7 has been described throughout the estrous cycle in the rat [50]. Similar to the rat, a differential regulation of claudins 1-4, occludin, as well as the adherens junction proteins E-cadherin and beta-catenin has also been shown in the endometrium of ewes throughout cycling [51]. It has been shown that claudin-3 protein is upregulated by progesterone in uterine epithelial cells of ovariectomized mice [52] and claudin-5 is downregulated by estrogens in murine endometrial endothelial cells [53]. In the latter study, it was supposed that claudin-5 regulation may play a role in the development of uterine edema, possibly mediated by estrogen-induced expression of the vascular endothelial growth factor (VEGF). An estradiol-induced VEGF-effect on tight junctions has also been shown in the baboon uterine endometrium by increasing the microvascular paracellular cleft width [54].

Using primary culture of human endometrial epithelial cells, an upregulation of claudin-1, -3, -4 and -7 protein content by progesterone and an inhibition of this upregulation by estradiol was observed. Furthermore, the barrier function of the tight junction as measured by the transepithelial electrical resistance decreased under the influence of progesterone, but not estradiol, while the treatment did not affect the fence function as determined by BODIPY-sphingomyelin diffusion in the membrane [38]. From these studies it has been concluded that the barrier function regulating paracellular permeability of the tight junctions can be varied by hormonal changes to provide an adequate environment for successful fertilization and early embryo development. However, in another study claudin-3 expression was upregulated by both progesterone and estradiol in the human endometrial epithelial cell line ECC-1 [52], indicating a regulation of the protein which differs slightly from the regulation in primary human endometrial epithelial cells and rodents.

The hormonal regulation of endometrial gap junction connexins has been studied in a variety of species. Rodents revealed a similar expression pattern like humans showing Cx26 in the uterine epithelium and Cx43 in the stromal cells [45]. Here, it could be proven that Cx26 and Cx43 protein expression are induced by estrogen and suppressed by progesterone [55,56,57], the latter leading to a complete suppression of gap junction protein expression during the receptive phase of the endometrium [45]. Moreover, it was shown that Cx26 in the endometrial epithelium reveals a high sensitivity to the ratio of progesterone to estradiol, since Cx26 can be re-induced in the uterine epithelium by increasing estradiol levels despite high progesterone concentrations [57]. Experiments in estrogen receptor-alpha and -beta knockout mice indicated that this upregulation was mediated via the estrogen receptor-alpha [58]. The endometrial Cx26 shows not only a high sensitivity to estradiol, but also to the strong estrogen agonist diethylstilbestrol (DES) and to selective estrogen receptor modulators like tamoxifen or raloxifene, as well as to the phytoestrogen genistein. These compounds also act via the estrogen receptor since their effect was inhibited by simultaneous application of an antiestrogen [59]. In this study, it was proven that phytoestrogens are able to shift the endometrial gene program even at a relatively low dose, and that the Cx26 gene expression in the rat endometrium can serve as a model for biological activity of estrogens. It is known that phytoestrogens reveal multiple biological effects, including beneficial effects on osteoporosis, on the cardiovascular system and on menopausal symptoms, but it has to be taken into account that they may also shift the hormonal homeostasis, and thus cause severe reproductive tract disorders, including impaired fertility.

To sum up, the distribution and function of the junctional protein complexes are partly sensitive to hormonal regulation. These results are summarized in Table 2. Although the precise mechanisms underlying junctional regulation in the human endometrium are not fully understood yet, a precise regulation of the different junctional components seems to be important for cyclic remodeling of the endometrium and as a consequence for its function during pregnancy.

## 5. Cell–Cell Junctions during Implantation and Decidualization

During the luteal phase of the menstrual cycle, the human endometrium is transformed to the receptive state to allow adhesion and invasion of the trophoblast. For successful implantation, however, gene expression in the endometrium in addition is regulated by the implanting blastocyst by precisely synchronized embryo-maternal interactions [60,61,62]. Both compartments of the endometrium are involved in this process: on the one hand, the epithelium has to allow adhesion of the embryo and invasion through the epithelium, on the other hand the stromal cells must be transformed to decidua cells which regulate trophoblast invasion and provide the placental blood supply necessary for embryo nutrition. In humans, the distribution and function of the various junctional proteins are not fully understood yet, although their importance in the process of implantation and pregnancy is eminent. Since these early embryo-maternal interactions cannot be investigated in humans, numerous studies have been conducted in various animal species, most of them in mouse models.

### 5.1. Changes in Epithelial Junctions during Embryo Implantation

In the rodent, the blastocyst reaches the uterine lumen on 4.5 dpc in the mouse and on 5 dpc in the rat. Degradation of the luminal epithelium and start of trophoblast invasion is observed from 5.5 dpc (mouse) or 6 dpc (rat) onwards [61,63]. Freeze fraction studies revealed that strands of tight junctions expand during the preimplantation phase of pregnancy on the lateral membrane of the uterine epithelial cells in pregnant rats [64] and in pseudopregnant rabbits [65]. In the rat, ZO-1 has been localized along the apical region of lateral plasma membranes of uterine epithelial cells from day 1 to 6 pc. In these stages of pregnancy, the claudin-1 protein was co-localized with ZO-1 in the apical region of the lateral plasma membrane and revealed a strong increase on day 6 pc. In parallel, occludin expression, which was absent on day 1–3 pc, was strongly induced at the time of uterine receptivity on 6 dpc in this apical region [66]. It has been discussed that occludin in uterine luminal epithelium may interact with claudins to form tight junction connections that control the volume and composition of uterine luminal fluid at the time of implantation to facilitate embryo implantation [67]. This is supported by the finding that besides claudin-1 also claudin-4 proteins increased from day 1 to day 6 pc in the luminal epithelium of the rat endometrium [66,67]. While claudin-3 revealed a consistent strong staining during this early phase of pregnancy both in glandular and luminal epithelial cells in the rat endometrium [67], it was shown in mice that the subcellular localization of claudin-3 and -7 switched from an apical and basal distribution to a strongly apically localization on day 4.5 pc in the luminal epithelium [52,68]. In contrast, high amounts of claudin-10 protein were present in the glandular epithelium in mice, but this claudin was absent in the luminal epithelium during the preimplantation period [68]. In these studies, however, it was not proven that these changes are dependent on the presence of a blastocyst.

Desmosomes [69], hemidesmosomes [70] and adherens junctions [71,72] were described to decline in the preimplantation period, presumably facilitating trophoblast invasion through the epithelial barrier. However, when E-cadherin was conditionally knocked out in the uterus, those mice revealed an implantation failure supposable due to the impairment of blastocyst adhesion to the luminal epithelium [73].

Similar to the situation in humans, gap junction intercellular communication is suppressed in the rodent uterine epithelium during the receptive phase. Prior to implantation, however, Cx26 is induced locally restricted to the luminal epithelium of the implantation chamber [45,56]. This has been shown to be due to a local effect of the blastocyst, since this antigen as well as the corresponding transcripts were neither detected in the inter-blastocyst segments nor in pseudopregnant animals [45,56,74]. This specific blastocyst-mediated induction of Cx26 was shown to act via an estrogen receptor-independent pathway and could also be induced by a mechanical stimulus in the hormonally primed receptive endometrium [58]. Experiments with pseudo-pregnant uteri in organ culture revealed that an inflammatory cascade may be involved in this blastocyst-mediated up-regulation of Cx26 in the uterine epithelium [58]. Such a locally restricted induction of gap junction proteins by the blastocyst in the non-coupled receptive uterine epithelium has also been described in other mammalian species like the rabbit (Cx32) [75] and the ewe (Cx26) [76]. It has been proposed that the restricted expression of Cx26 in the epithelium of the implantation chamber regulates the controlled cell death of the uterine epithelium accompanying the implantation process in the rodents [77]. However, the specific role of this precisely spatially and timely regulated connexin suppression and induction for implantation still remains to be elucidated. It was shown that embryo implantation was impaired by injection of a non-specific gap junction channel blocker [78], however, since this compound not only blocked epithelial but also stromal gap junction channels, a specific role for the epithelial induction of gap junctional communication could not be proven in this study.

The crucial function of junctional components in the uterine epithelium might be to control epithelial permeability and thus the uterine milieu as well as regulating trophoblast adhesion to and penetration through the epithelial lining, whereby the various junctional proteins may exhibit different functions. There is evidence that tight junctions are the only junctional complexes that are maintained during the implantation window. The tight connection of the epithelial cells may maintain an optimal uterine micro milieu for the developing blastocyst during the sensitive phase of implantation [79,80]. In contrast, components of adhesion and gap junctions decrease during preimplantation, possibly to facilitate trophoblast invasion through the epithelial barrier, though some of them, like E-cadherin, may be necessary for successful blastocyst attachment.

### 5.2. Changes in Stromal Junctions during Decidualization

In preparation for embryo implantation, not only does the uterine epithelium have to differentiate into a receptive state to allow adhesion and invasion of the trophoblast, but the endometrial stromal cells also undergo a complex differentiation process. They transform to decidual cells, which regulate trophoblast invasion, may be involved in the selection of competent embryos, and, moreover, support angiogenesis to build up an extensive vascular network, which is essential for placental blood supply and successful embryonic development [4,81]. Thus, an adequate decidualization process is indispensable for successful embryo implantation and development [82]. During this process, the endometrial stromal cells undergo phenotypic changes reminiscent of mesenchymal-epithelial transition leading to epitheloid cells [83,84,85], accompanied by changes in expression and localization of numerous cell–cell contact proteins.

In the pre-decidual cells of the human luteal phase endometrium, up to now, only the gap junction protein Cx43 has been described, while there is no knowledge about junctional proteins in the human decidual cells during the early stages of pregnancy. When decidualizing human endometrial stromal cells in vitro, it has been shown that the expression of originally epithelial proteins, including beta-catenin, E-cadherin and ZO-1, is redistributed to the decidualized stromal cells [86], supporting the mesenchymal-epithelial transition. An important role of this induction of E-cadherin for the decidualization process has been demonstrated in mice that lack uterine E-cadherin. In these mice, no decidual response could be observed when decidualization was artificially induced [73].

In rodents, decidualization of endometrial stromal cells is induced with the start of the implantation process. First, the stromal cells surrounding the implantation chamber differentiate to the avascular primary decidual zone, encapsulating the implanting embryo [87]. Here, it has been shown that tight junction proteins are induced during this mesenchymal-epithelial transition of the endometrial stromal cells. However, freeze-fracture studies revealed incomplete tight junctions in the primary decidual zone which were supposed to function as semipermeable barriers to allow the transport of large molecules paracellularly through this compact zone to the developing embryo [88]. Meanwhile, the tight junction proteins occludin, ZO-1, ZO-2 and claudin-1 were demonstrated to form associated complexes in these decidual cells of the primary decidual zone on day 6 pc, forming a barrier surrounding the embryo concurrently with the loss of the adjacent luminal epithelium [87]. Moreover, a strong induction of claudin-10 was observed in the primary decidual cells already on day 4.5 pc—thus, prior to trophoblast invasion—and expanded to the secondary decidua thereafter [68]. From 6.5 dpc onwards, additionally, an intense staining for the claudin-3 protein appeared in the cells of the secondary decidua [52,68], which was co-localized with the endothelial cell marker CD31 towards the mesometrial part of the implantation site [68]. Since Claudin-3 has been described as taking part in building up the blood-brain barrier in endothelia of the central nervous system [89], this protein distribution also could be involved in protecting the implantation site from immunoreactive substances originating from the maternal circulation.

To determine which component of a blastocyst is necessary to induce expression of tight junctional proteins in the decidua, Wang and colleagues examined the expression of various proteins of the tight junction complex in the presence of either a normal blastocyst, trophoblast vesicles or isolated inner cell mass [87]. While blastocysts and trophoblast vesicles induced a similar expression of tight junctional proteins, isolated inner cell mass failed to initiate such a reaction. From these findings, the authors concluded that the trophectoderm appears to be the stimulus for the establishment of the barrier surrounding the embryo.

Besides the induction of these junctional proteins, the decidual cells are also extensively connected by gap junctions. In human, baboon and rodent endometrium, Cx43 is the dominantly expressed gap junction subunit in the stromal compartment [90]. In rodents, stromal Cx43 is suppressed during the receptive phase and increases considerably during decidualization starting in the primary decidual zone and then spreads out throughout the implantation chamber with ongoing decidualization [45,56]. In rats, but not in mice, in parallel Cx26 is induced in the decidual cells [45]. The presence of Cx43 in the decidua is important for the transformation of stromal cells into the compact decidua, as well as for the formation of new maternal blood vessels in the stromal compartment, which is critical for the establishment and maintenance of pregnancy. This has been proven in mice displaying a conditional deletion of Cx43 in the endometrial stromal cells. This deletion inhibited the transformation of the endometrial stromal cells to decidual cells, concomitant with induction of apoptosis [91], and impaired decidual angiogenesis, resulting in the arrest of embryo growth and early pregnancy loss [92]. Decidual angiogenesis may also be influenced by Cx43 in the uterine vascular endothelium which is involved in cell signaling regulation of uterine blood flow [93]. The important role of this intercellular communication for the decidualization process has been confirmed in human endometrial stromal cells in vitro. Here, knockdown of Cx43 or pharmacological disruption of gap junctional communication impaired decidualization as substantiated by inhibition of secretion of prolactin and VEGF as well as of the expression of markers for mesenchymal-epithelial transition [86,94]. In contrast, overexpression of Cx43 in human endometrial stromal cells led to an upregulation of markers for mesenchymal-epithelial transition as well as of VEGF and ZO-1. In parallel, the expression of N-cadherin as an indicator of epithelial-mesenchymal transition was inhibited [86].

Summing up these findings, there is a considerable induction of various junctional proteins during decidualization which build a selective barrier towards the embryo after breakdown of the epithelial barrier. These proteins play an important role in paracrine signaling within the decidua to sustain differentiation and to support angiogenesis in the maternal compartment as a prerequisite for nutrition of the growing embryo. The clinical significance of these observations is supported by findings that Cx43 levels are reduced in the decidua of women with recurrent early pregnancy loss [95] and by the fact that the anti-malarial medication mefloquine, which blocks Cx43 gap junctions, is associated with an increased risk of spontaneous abortion [96]. Moreover, impaired endometrial decidualization is increasingly attributed to pathophysiological conditions associated with reduced fecundity and pregnancy complications. These include endometriosis, polycystic ovary syndrome, recurrent miscarriage, pre-eclampsia, and preterm birth [81,97,98,99,100,101].

In conclusion, there is a precise temporal and spatial regulation of various junctional proteins in the epithelial, as well as the stromal, compartment of the endometrium during the implantation process (summarized in Table 3). Though the definite role of these proteins in this context has not been deciphered in detail yet, its precise regulation assumes a considerable role in endometrial function, and disruption of these patterns, were shown to lead to impairment of the implantation process or of placental and fetal development.

## 6. Direct Cell–Cell Interactions in Endometrial Pathophysiology

### 6.1. Endometriosis

Endometriosis is characterized by endometriotic tissue growing outside the uterine cavity, affecting 10–15% of women of reproductive age and even up to 50% of women seeking infertility treatment. Although it is a benign endometrial disease, it leads to severe clinical symptoms such as abdominal pain and subfertility [102]. One cause for the ectopic colonization and growth of endometrial tissue may origin in an inappropriate differentiation of the endometrial cells leading to an increase in adhesiveness and invasiveness of the endometriotic tissue. This impairment of differentiation may affect the epithelial-to-mesenchymal or mesenchymal-to-epithelial transition in the endometrial tissue [103], which physiologically is accompanied by a highly regulated expression pattern of intercellular junctional complexes. This has been supported by a morphometric study showing that tight junctions were missing or disrupted in endometrioma compared to eutopic endometrium [39]. In a more recent study, microarray analyses revealed an upregulation of transcripts of JAM-B and JAM-C and of claudin-1, -5 and -11 and a downregulation of ZO-3, occludin and claudin-3, -4 and -7 in peritoneal endometriotic lesions compared to the corresponding eutopic endometrium [104]. However, these observations were not validated by PCR or immunohistochemical staining. In accordance with these findings, Pan and colleagues noted a significantly lower expression of claudin-3 and -4 in ovarian endometrioma compared to eutopic endometrium originating from patients with endometriosis and from healthy controls on mRNA and protein level [39]. In contrast, immunohistochemical analysis showed a decreased staining for claudins-1 and -5 in epithelial cells of peritoneal endometriotic lesions [36].

One hallmark of epithelial-to-mesenchymal transition is the functional loss of E-cadherin expression in epithelial cells. A reduction of E-cadherin, as well as alpha- and beta-catenin, expression in peritoneal [105,106,107] and ovarian endometriosis [108] compared with the eutopic endometrium has been described. This is supported by studies demonstrating that E-cadherin-negative epithelial cells were increased in peritoneal endometriosis compared with eutopic endometrium and that in vitro, E-cadherin-negative, but not E-cadherin-positive epithelial cells, showed invasive growth [109]. Thus, the loss of E-cadherin expression could constitute a crucial mechanism in the pathogenesis of endometriosis by increasing the invasiveness of endometriotic cells.

Moreover, the inappropriate differentiation of endometrial tissue in endometriosis patients is correlated with an aberrant expression of gap junction connexins. In the eutopic endometrium of women with endometriosis a significant decrease in Cx43 has been described, which correlated with a decrease in physiological cell–cell coupling, while no changes in Cx26 were observed [110]. In parallel to the decrease in Cx43 expression and cell coupling, in vitro decidualization was impaired in these cells, supporting a role of impaired decidualization in the pathogenesis of endometriosis. An aberrant allocation of connexin proteins has also been described in ectopic endometrial lesions. Here, Cx43 expression was enhanced in the endometriotic glands, whereas the number of patients exhibiting Cx26, typical for human uterine epithelium cells, was strongly reduced, and Cx32 was not detectable [111]. Moreover, Cx43, which is located in the stromal cells of healthy patients, was not present in this tissue compartment in endometriotic lesions. Similar results were obtained in the eutopic endometrium of baboons in which endometriosis had been experimentally induced. Here, a loss of Cx26 and Cx32 in the epithelium and an up-regulation of Cx26 in the stromal cells have been observed [112].

Taken together, the above-described alterations in direct cell–cell interaction may contribute to a change in the differentiation program of both the epithelial and stromal compartment of the endometrium. These alterations are summarized in Table 4. Although endometriosis is considered a ‘benign’ disease, it resembles the biologic behavior of malignant cells [113,114], and a change in the expression of various junctional proteins may support the invasive properties of this tissue and may facilitate its growth at ectopic localizations. Moreover, these impairments may also contribute to endometriosis-associated infertility.

### 6.2. Endometrial Carcinoma

Endometrial carcinoma is one of the most frequently diagnosed gynecological malignancies [115]. Based on clinical and histopathological criteria, it is classified in two subtypes. Endometrioid adenocarcinoma (Type I), which accounts for about 80% of cases, is low-grade, estrogen-dependent and usually associated with complex and atypical endometrial hyperplasia, whereas type II endometrial carcinoma include serous papillary and clear cell types and is more aggressive and estrogen-independent [116,117]. For the general pathogenesis and progression of cancer, changes in cell–cell contacts have been described to play an essential role [118,119,120]. They may act via their intercellular communication functions, but may also exhibit their effect independently from these roles since they may be involved in signal transduction regulating gene expression [121,122].

Also, in the pathogenesis and progression of endometrial cancer, changes in cell–cell junctions have been described. A morphological disruption of tight junctions was observed in endometrial adenocarcinoma, but not in atypical hyperplastic endometrium [33]. In parallel, altered claudin expression has been described in the malignant endometrial tissues in this study. In endometrial adenocarcinoma claudin-3 and -4 mRNA and protein increased with the clinicopathologic features of the tissue, progressing from simple to complex and from atypical hyperplasia to endometrioid carcinoma [33]. Since the upregulation of claudins was already visible in atypical hyperplasia but the morphological degeneration of the tight junctions only in endometrioid carcinoma, it has been supposed that the elevated claudin level precedes the disruption of tight junctions. A significant upregulation of claudin-3, -4 and -7 compared to normal endometrial cells has also been found in primary culture of uterine serous papillary tumor cells, the most aggressive kind of estrogen-independent type II endometrial carcinoma [123]. In contrast, claudin-5 was significantly decreased in these tumor cells. Beyond this, the presence of different claudin subtypes may differ in the different types of endometrial cancer. By evaluating immunohistochemical scores, low claudin-1 and high claudin-2 protein contents were found in hyperplasia and endometrioid adenocarcinoma (type I), whereas in seropapillary adenocarcinoma (type II), high claudin-1 and low claudin-2 levels were detected [35].

Since claudin-4 and, with a lesser affinity, also claudin-3 act as epithelial receptors for *Clostridium perfringens* enterotoxin (CPE) [124,125,126], probably mediated by binding to the free second extracellular loop of claudins, they may constitute suitable targets for this anti-cancer drug which may be effective also in tumor cells refractory to chemotherapy [127,128]. This is supported by the finding that the cytotoxicity of CPE was even enhanced in an endometrial adenocarcinoma cell line after upregulation of claudin-3 and -4 [38]. The emerging evidence of the involvement of claudins in the pathogenesis of endometrial carcinoma of various subtypes is consistent with findings concerning the pathogenic role of claudins in a variety of other tumors such as in breast, gastric, pancreatic and prostate cancers [129].

In regard to adhesion contacts, the role of the two adhesion molecules E-cadherin and beta-catenin in the carcinogenesis of endometrial carcinoma has been extensively studied, and the expression of these proteins is discussed as a prognostic marker. Although varying in detail, most studies are consistent that low E-cadherin expression correlates with increasing aggressiveness, poor differentiation, and deep myometrial invasion of the carcinoma [130,131,132,133,134,135,136,137,138,139]. In accordance, E-cadherin was found to be more often and prominently expressed in endometrioid adenocarcinoma than in serous papillary or clear cell tumors [131,139,140], and a high E-cadherin level has been associated with reduced mortality, disease progression, and disease recurrence rate and thus is associated with a better prognosis [141]. However, a correlation between clinicopathological factors and the score or intensity of E-cadherin immunohistochemical staining of endometrial carcinoma could not be confirmed in another study [142], advising to carefully control the classical clinicopathologic criteria in regard to E-cadherin expression. Further insights on the role of E-cadherin in endometrioid adenocarcinoma are constantly emerging. For example, in endometrioid endometrial carcinoma the expression of the E-cadherin suppressor Snail was found to be negatively correlated with E-cadherin expression [130] and was correlated with abnormal E-cadherin expression in metastases of this tumor [132].

Also for beta-catenin, a decreased level has been demonstrated with increasing grading of endometrial carcinoma [143]. Moreover, β-catenin gene (*CTNNB1*) mutations led to decreased cell–cell adhesion and have been reported in about 15% of endometrioid carcinomas [144,145]. Since beta-catenin is a transcription factor that is involved in the Wnt signal transduction pathway, which in turn is crucial for carcinogenesis, it may exhibit its effect via this signaling pathway [135].

In addition to the roles of tight and adherens junctions described above, there is substantial evidence that an interruption of gap junctional communication or the aberrant expression of connexins constitutes one important step in carcinogenesis [118]. In endometrial hyperplasia and carcinoma, the amount of Cx26 and Cx32 in the uterine epithelium, as well as Cx43 in the endometrial stromal cells, and, as a consequence, gap junctional communication, is reduced and/or aberrantly localized [146,147]. These studies showed that during endometrial carcinogenesis, loss of gap junctional intercellular communication may occur at relatively early stages. A correlation between a reduced connexin expression and the progression of cancer was supported by the observation that activation of the estrogen receptor-alpha by estrogen, which is a primary etiological factor associated with the development of endometrial hyperplasia and adenocarcinoma, reduced gap junctional intercellular communication, and expression of Cx26 and Cx32 in endometrial carcinoma cells [148]. Interestingly, numerous studies have demonstrated that not only the amount of connexins, but also their localization, may effect tumor growth. Connexins may thus act by other mechanisms than by functional coupling of cells. A mutated form of Cx43, revealing a change in protein sequence of the second extracellular region of Cx43 which prevented incorporation of the protein into the plasma membrane, did not decrease its ability to inhibit the growth of tumor cells in vitro [149]. Thus, regulation of cellular growth by Cx43 does not necessarily require well-functioning gap junctions. This has been affirmed by several reports describing the tumor-suppressing properties of Cx43 and Cx26 in the absence of functionally coupled channels, possibly by regulating key genes involved in tumor growth [150,151].

The junctional proteins regulated in endometrial carcinogenesis are summarized in Table 5.

In conclusion, the different cell–cell contact proteins may exhibit considerable effects on the pathogenesis of endometrial cancer. Besides ensuring cohesion of a healthy cell structure, they may regulate signaling pathways involved in the pathogenesis and progression of endometrial cancer, and thus may represent promising tools for diagnostic and therapeutic approaches in cancer treatment.

## 7. Conclusions

Direct cell–cell junctions are highly specific and precisely regulated during the physiological changes in the endometrium as well as in pathological conditions. Despite their specific function in cell–cell interaction they also may regulate signaling pathways, thereby influencing gene expression in the different compartments of the endometrial tissue. Moreover, besides close interactions of various proteins within the complex structure of the same junctions and with components of the cytoskeleton, insights are increasing about close relationships between the proteins of different junctional complexes. Thus, a close interaction of the components of the different cell–cell junctions might also play an important role in the different physiological conditions of the endometrium. The number of components building involved in these junctions and their interactions has grown considerably in recent years, and up to now, only some of them have been analyzed in the endometrium. An adequate expression of the different junctional proteins in the endometrium is indispensable, since genetic defects and dysregulation of these interactions can cause different diseases, and may impair the implantation reaction and embryonal or placental development resulting in phenomena like preeclampsia or fetal growth restriction. Still, many questions remain concerning the various functions of junctional proteins and their interactions. Extending our knowledge of these essential functions in endometrial physiology and pathogenesis will provide closer insight in female reproductive health.

## Figures and Tables

**Table 1:** Distribution of junctional proteins in cycling human endometrium.

Junctional Component	Analyzed Parameter	Localization	Regulation	Reference
Claudin-1	mRNA		Not regulated	[36]
	Protein	GE	Upregulated in SP	[35]
Claudin-3	mRNA		Upregulated in mid SP	[36]
	Protein	GE	Not regulated	[33,39]
Claudin-4	mRNA		Upregulated in mid SP	[35,36]
	Protein	GE	Not regulated	[33,35,37,39]
Claudin-5	mRNA		Not regulated	[36]
	Protein	GE	Upregulated in SP	[35]
Claudin-7	mRNA		Upregulated in mid SP	[36]
ZO-1	mRNA		Not regulated	[37]
	Protein	GE	Not regulated	[37]
JAM-1	mRNA		Not regulated	[41]
	Protein	GE	Not regulated	[41,42]
Desmoplakin 1/2	Protein	GE (functionalis)	Change of localization	[37]
Desmoglein 2	Protein	GE (functionalis)	Change of localization	[37]
E-cadherin	mRNA		Not regulated	[37]
	Protein	GE	Downregulated in SP	[37]
β-Catenin	Protein	GE	Downregulated in SP	[37]
Cx26	Protein	LE/GE	Downregulated in SP	[44]
Cx32	Protein	GE	Downregulated in mid SP	[44]
			Upregulated in early SP/Downregulated in late SP	[46]
Cx43	Protein	Stromal cells	Downregulated in SP	[44]
			Upregulated in SP	[47]

GE = glandular epithelium; LE = luminal epithelium; SP = secretory phase.

**Table 2:** Hormonal regulation of junctional proteins in the endometrium.

Junctional Component	Species	Localization	Regulation	Reference
Claudin-1	Human	Primary hEEC	Upregulated by P/inhibited by E	[38]
	Rat	Epithelial cells	Change in localization	[50]
Claudin-3	Human	Primary hEEC	Upregulated by P/inhibited by E	[38]
	Rat		Change in localization	[50]
Claudin-4	Human	Primary hEEC	Upregulated by P/inhibited by E	[38]
Claudin-5	Mouse	Endothelial cells	Downregulated by E	[53]
	Rat	Epithelial cells	Change in localization	[50]
Claudin-7	Human	Primary hEEC	Upregulated by P/inhibited by E	[38]
	Rat	Epithelial cells	Change in localization	[50]
Zo-1	Rat	Epithelial cells	Not regulated	[50]
Occludin	Rat	Epithelial cells	Upregulated by E	[50]
Cx26	Rat	Epithelial cells	Upregulated by E/inhibited by P	[55,56,57]
Cx43	Rat	Stromal cells	Upregulated by E/inhibited by P	[55,56,57]

hEEC = human endometrial epithelial cells; P = progesterone; E = estrogen.

**Table 3:** Regulation of junctional proteins during implantation and decidualization.

Junctional Component	Species	Localization	Regulation	Reference
Claudin-1	Rat	Epithelial cells	Increased on 6 dpc	[66]
Claudin-3	Mouse	Decidual cells	Induced on 6.5 dpc	[52,68]
			Change of localization on 4.5 dpc	[68]
Claudin-4	Rat	Epithelial cells	Increase from 1–6 dpc	[67]
Claudin-10	Mouse	Decidual cells	Induced on 4.5 dpc	[68]
Occludin	Rat	Epithelial cells	Induced on 6 dpc	[66]
Cx26	Rat	Epithelial cells	Induced on 5 dpc	[45]
		Stromal cells	Induced on 6 dpc	[45]
	Mouse	Epithelial cells	Induced on 4.5 dpc	[58]
Cx43	Rat	Decidual cells	Increased during decidualization	[45]
	Mouse	Decidual cells	Increased during decidualization	[58]

Dpc = days *post coitum*.

**Table 4:** Regulation of junctional proteins in endometriosis.

Junctional Component	Analyzed Parameter	Regulation	Reference
Claudin-1	mRNA	Upregulated in peritoneal lesions	[104]
	Protein	Downregulated in peritoneal lesions	[38]
Claudin-3	mRNA	Downregulated in peritoneal lesions	[104]
		Downregulated in ovarian endometriomata	[39]
	Protein	Downregulated in ovarian endometriomata	[39]
Claudin-4	mRNA	Downregulated in peritoneal lesions	[104]
		Downregulated in ovarian endometriomata	[39]
	Protein	Downregulated in ovarian endometriomata	[39]
Claudin-5	mRNA	Upregulated in peritoneal lesions	[104]
	Protein	Downregulated in peritoneal lesions	[38]
Claudin-7	mRNA	Downregulated in peritoneal lesions	[104]
Claudin-11	mRNA	Upregulated in peritoneal lesions	[104]
Jam-B	mRNA	Upregulated in peritoneal lesions	[104]
Jam-C	mRNA	Upregulated in peritoneal lesions	[104]
Zo-3	mRNA	Downregulated in peritoneal lesions	[104]
E-Cadherin	Protein	Downregulated in peritoneal lesions	[105,106]
		Not regulated in endometriosis	[107]
	mRNA	Downregulated in ovarian endometriomata	[108]
α-Catenin	Protein	Downregulated in peritoneal lesions	[105]
	mRNA	Downregulated in ovarian endometriomata	[108]
β-Catenin	Protein	Downregulated in peritoneal lesions	[105,107]
	mRNA	Downregulated in ovarian endometriomata	[108]
Cx26	Protein	No regulation in eutopic endometrium *	[110]
		Downregulated in peritoneal lesions	[111]
Cx43	Protein	Downregulated in eutopic endometrium *	[110]
		Downregulated in peritoneal lesions	[111]

* = of endometriosis patients.

**Table 5:** Regulation of junctional proteins in endometrial cancer.

Junctional Component	Analyzed Parameter	Tumor Staging	Regulation	Reference
Claudin-1	Protein	Type II (USPC)	Upregulated	[35]
Claudin-2	Protein	Type II (USPC)	Downregulated	[35]
Claudin-3	mRNA	Type I	Upregulated	[33]
		Type II (USPC)	Upregulated	[123]
	Protein	Type I	Upregulated	[33]
Claudin-4	mRNA	Type I	Upregulated	[33]
		Type II (USPC)	Upregulated	[123]
	Protein	Type I	Upregulated	[33]
Claudin-5	mRNA	Type II (USPC)	Downregulated	[123]
E-Cadherin	Protein	Type I/Type II	Downregulated during dedifferentiation	[130,131,132,133,134,135,136,137,138,139]
β-Catenin	Protein	Type I/Type II	Downregulated during dedifferentiation	[143]
Cx26	mRNA	Type I	Downregulated	[146]
	Protein	Type I	Downregulated	[146,147]
Cx32	mRNA	Type I	Downregulated	[146]
	Protein	Type I	Downregulated	[146,147]
Cx43	mRNA	Type I	Downregulated	[146]
	Protein	Type I	Downregulated	[146,147]

USPC = uterine serous papillary carcinoma.

## Abbreviations

CPE	*Clostridium perfringens* enterotoxin
Cx	Connexin
dpc	Days *post coitum*
E	Estrogen
GE	Glandular epithelium
hEEC	Human endometrial epithelial cells
IVF	*In vitro* fertilization
JAM	Junction adhesion molecule
LE	Luminal epithelium
LIF	Leukemia inhibitory factor
MUPP	Multi-PDZ domain protein
P	Progesterone
pc	*Post coitum*
PCR	Polymerase chain reaction
SP	Secretory phase
USPC	Uterine serous papillary carcinoma
VEGF	Vascular endothelial growth factor
ZO	Zonula occludens
